# Struvite-Driven Integration for Enhanced Nutrient Recovery from Chicken Manure Digestate

**DOI:** 10.3390/bioengineering11020145

**Published:** 2024-01-31

**Authors:** Seyyed Ebrahim Mousavi, Bernard Goyette, Xin Zhao, Cassandra Couture, Guylaine Talbot, Rajinikanth Rajagopal

**Affiliations:** 1 Sherbrooke Research and Development Center, Agriculture and Agri-Food Canada, 2000 College Street, Sherbrooke, QC J1M 0C8, Canada; ebrahim.mousavi65@gmail.com (S.E.M.); bernard.goyette@agr.gc.ca (B.G.); cassandra.couture@usherbrooke.ca (C.C.); guylaine.talbot@agr.gc.ca (G.T.); 2 Department of Animal Science, McGill University, 21111 Lakeshore Road, Saint Anne De Bellevue, QC H9X 3V9, Canada; xin.zhao@mcgill.ca; 3 Department of Biology, Université de Sherbrooke, 2500 Bd de l’Université, Sherbrooke, QC J1K 2R1, Canada

**Keywords:** ammonia inhibition, chicken manure digestate, heavy metal removal, nutrients recovery, struvite production

## Abstract

This study investigated the synergistic integration of clean technologies, specifically anaerobic digestion (AD) and struvite precipitation, to enhance nutrient recovery from chicken manure (CM). The batch experiments were conducted using (i) anaerobically digested CM digestate, referred to as raw sample (RS), (ii) filtered digestate sample (FS), and (iii) a synthetically prepared control sample (CS). The research findings demonstrated that the initial ammonia concentration variations did not significantly impact the struvite precipitation yield in the RS and FS, showcasing the materials inertness process’s robustness to changing ammonia concentrations. Notably, the study revealed that the highest nitrogen (N) recovery, associated with 86% and 88% ammonia removal in the CS and FS, was achieved at pH 11, underscoring the efficiency of nutrient recovery. The RS achieved the highest nitrogen recovery efficiency at pH 10, at 86.3%. In addition, the research highlighted the positive impact of reducing heavy metal levels (Zn, Cu, Pb, Ni, Cd, Cr and Fe) and improving the composition of the microbial community in the digestate. These findings offer valuable insights into sustainable manure and nutrient management practices, emphasizing the potential benefits for the agricultural sector and the broader circular economy. Future research directions include economic viability assessments, regulatory compliance evaluations, and knowledge dissemination to promote the widespread adoption of these clean technologies on a larger scale. The study marks a significant step toward addressing the environmental concerns associated with poultry farming and underscores the potential of integrating clean technologies for a more sustainable agricultural future.

## 1. Introduction

Today, the world has over 23 billion poultry, which represents about three birds per person on the planet [1]. The production of chicken meat, which is the most consumed source of animal protein, has been growing steadily over the past 50 years, with an annual rate of approximately 5%, compared to only 1.5% for beef and 3.1% for pork [2]. This high volume of production results in a huge amount of chicken manure (CM). Poorly managed manure can cause considerable environmental damage and is known to trigger foodborne and animal disease outbreaks.

The challenges associated with CM include greenhouse gases (GHGs), odor and ammonia emissions, and water pollution from inorganic (nitrogen (N), phosphorus (P), and potassium (K)) and organic pollutants (chemical oxygen demand (COD), antibiotics, and pathogens) [3]. The treatment of these wastes has recently received greater attention due to the challenges they pose, including their high organic and nutrient contents, as well as high contents of nitrogen and phosphorus. Excess nitrogen in CM is a major contributor to environmental pollution through the release of nitrates into surface and groundwater and the emission of ammonia into the atmosphere. A wide range of treatment technologies have been used to treat CM, such as anaerobic digestion, direct combustion, extrusion, and incineration [4,5,6,7]. Among these technologies, the use of agricultural organic waste for biogas production through anaerobic digestion (AD) is an effective means for waste management, pollution mitigation, renewable energy use, and GHG reduction [4].

Despite the effectiveness of AD, the use of this technique for CM has historically been challenging because of two major problems. First, poultry do not have urinary tracts and their waste is excreted at approximately 25% total solids (TS). In addition, their manure is mixed with feathers and bedding materials used in poultry houses, resulting in a high solids content (40–60% TS). For a successful AD operation, the CM should be diluted (five to eight times) with water to about 7–10% TS. This dilution will create a greater removal problem and increase water usage. Secondly, and more importantly, a high ammonia concentration in CM will lead to an inhibition of methanogenic archaea in the AD process [8]. As indicated, the ammonium–nitrogen concentrations in AD digestate are in the range of 0.8–5.0 g/L [9,10]. Similarly, phosphorus in CM could reach up to 1.21 g/L [11]. The development of appropriate strategies to avoid ammonia inhibition in the AD process is a major concern for maximizing biogas production. In addition, the integration of AD and the simultaneous recovery of nitrogen and phosphorus offer great potential for solving the CM problem.

Various techniques have been developed in recent decades to recover nutrients, such as N and P, from animal waste [12,13]. Among these techniques, chemical precipitation has shown promise because it can handle a wide range of nutrient concentrations, making it ideal when dealing with high ammonia waste, such as CM. Struvite (ammonium magnesium phosphate hexahydrate [MgNH_4_PO_4_·6H_2_O]) precipitation is one such technique that not only efficiently recovers N and P from raw or digested materials but also harnesses these nutrients as a valuable resource for agricultural land. Struvite is considered a slow-release fertilizer, which is suitable for cultivation without the risk of burning plant roots [14]. As a soft mineral, it can also resist leaching by rain [15]. In addition, the nitrogen in struvite is a more stable form for plant uptake than traditional manure application. It has been reported that if manure is applied to the soil surface, up to 25% of the ammonium nitrogen can be lost after two days and 75% or more after one month of application [16]. Based on the chemical composition of struvite, it contains approximately 13% phosphorus, 6% nitrogen, and 10% magnesium [17], making it an effective fertilizer for field crops as well as for orchards, potted plants, and ornamentals. Numerous studies have examined the effectiveness of struvite fertilizer applications on various field crops, such as corn [18], wheat [19], and winter barley [20].

The use of struvite as a fertilizer on farms requires additional post-production steps, including size grading for different applications as well as transportation and handling. For this reason, particle size is an important parameter in the struvite production process that has not been studied extensively to date, especially as a by-product of internal manure processing on farms where a specific particle size may be desired [21,22,23]. Although extensive research has been conducted on the integration of struvite precipitation into the anaerobic digestion of dairy and swine manure [24,25,26,27,28], a limited number of studies have been conducted on this integration into CM [29,30,31]. The management of chicken manure using CleanTech solutions involving anaerobic digestion and struvite precipitation faces several challenges [12]. The variability in feedstock composition, influenced by factors like diet and bird age, can impact the process efficiency and product quality. Process optimization, including the pH, temperature, and nutrient control, is critical for optimal performance. Struvite scaling may occur, reducing the process efficiency and increasing maintenance costs. Managing excess nutrients recovered as struvite is essential to avoid environmental issues. Furthermore, the economic feasibility of this approach depends on factors such as the scale, funding availability, incentives, and market value of the end products.

Given the prevailing challenges, it is crucial to conduct further research for the refinement of the integration under consideration. Thus, this study aimed to assess the feasibility of integrating the struvite precipitation technique with the anaerobic digestion (AD) process. The primary goal was to enhance nutrient recovery from chicken manure digestate, ultimately producing a high-quality fertilizer tailored for agricultural use. Additionally, the critical parameters of this process, including the pH, ammonia concentration, and molar ratio of the components, were investigated. Furthermore, recognizing the potential positive impacts of integrating manure anaerobic digestion with struvite precipitation on heavy metal removal and the improvement of the microbial community composition in the digestate, this study also examined the effects on these two factors.

## 2. Materials and Methods

### 2.1. Batch Experimental Setup

A series of 250 mL batch experiments were carried out with (i) the raw sample (RS) corresponding to the three different anaerobically digested CM samples (digestate), (ii) the filtered digestate sample (FS) corresponding to the filtrate after filtration, and (iii) the control sample (CS), which was synthetically prepared. The RS was designated as undiluted leachate obtained from a laboratory anaerobic digester (20 L working volume) processing chicken manure (TS: 68%; NH_3_: 8 g/L) and operated at 20 ± 1 °C for 282 d over four batches (~70-d/batch) at an OLR of 8.78–4.3 gVS/L/d, as previously described by [32]. The FS was subjected to solid–liquid separation, as described in Section 2.2, and the CS was synthetically prepared (using Fisher ACS grade MgCl_2_·6H_2_O, KH_2_PO_4_ and (NH_4_)_2_SO_4_, and distilled water), mainly to compare the performance of the FS. To evaluate the influence of the different parameters, including the pH, Mg:P:N molar ratio, initial ammonia concentration, and presence of solids on struvite precipitation, a series of experiments were performed, as shown in Table 1.

Firstly, a series of experiments were performed with the FS to study the feasibility of struvite precipitation from the CM digestate. Then, a comparative study was carried out with a series of CS solutions (all at different concentrations). In order to reduce the possible impact of suspended solids on precipitation, the experiments began with the FS solution and not the RS. In addition, to investigate whether a high concentration of ammonia in the digestate might hinder precipitation or cause the precipitation of by-products, different concentrations of the two solutions (FS and CS) were prepared, as shown in Table 1. Once these preliminary experiments had been completed, the experiment with a 100% pure digestate from the RS100 was carried out and compared with the FS100 and CS100. There was no need to use lower concentration RS solutions (i.e., RS10, RS25, RS50) since RS100 had the expected struvite precipitation rate (as well as ammonia removal efficiency) compared to CS and FS solutions. Next, experiments R2 to R10 were designed to study the impact of the pH and initial molar ratio in order to find the ideal conditions for precipitation. Experiments R2 to R9 were different experiments with a variation of one parameter compared to the other experiments. Table 1 explains the conditions in each experiment.

To investigate the impact of the initial ammonia concentrations, different solutions with different initial total ammoniacal nitrogen (TAN) concentrations were prepared with distilled water, namely 10%, 25%, 50%, and 100% (pure digestate), corresponding to a digestate/water ratio of 1:9, 2.5:7.5, 5:5, and 10:0, respectively.

All the experiments were performed at room temperature (20–21 °C). For each batch test, all the required materials were mixed at a requisite molar ratio and then the pH was adjusted to the desired value, ranging from 8 to 11, in the different experiments using 1 N and 5 N NaOH and 1 N HCl. The solutions were pre-mixed for 5 min to achieve a stable pH and temperature and to ensure homogeneity. Then, they were mixed at 150 RPM for 60 min, which included a pH adjustment, time for reaction, and precipitation. Then, the produced struvite was separated using a 0.45 micrometer membrane vacuum filtration technique for the CS and FS. Due to the high solids content of the RS solutions, membrane filtration was not applicable, and therefore the centrifugation technique was used for the RS solutions. The precipitates (struvite) were dried at 35 °C for 24 h for the CS and FSs and 48 h for the RSs. The supernatants were collected and labeled for further characterization of the nutrients and heavy metals. Preliminary feasibility experiments on the CS and FS at different dilution factors were conducted at pH 9, with a Mg:P:N molar ratio of 1:1:1 and a total mixing time of 60 min.

### 2.2. Digestate Sample Preparation

The anaerobically pretreated CM digestate used in this study was obtained from our ongoing laboratory-scale two-stage AD at a low temperature (20 ± 1 °C), whose operating cycle length was maintained at approx. 70 d/batch, as previously described by [32]. The digestates from three different 20 L digesters (in duplicate) were mixed in a vessel and 20 L were collected as a representative sample in a plastic container, which was stored at 4 °C for subsequent physicochemical characterizations. For the study of struvite precipitation, this digestate was referred to as the RS and part of it was subjected to solid–liquid separation to obtain the FS. This was done mainly to study the impact of the TS on struvite production. Solid separation was carried out by centrifugation at 14,000 rpm for 30 min. The physicochemical characteristics of the two samples (RS and FS) are presented in Table 2.

### 2.3. Materials and Analytical Methods

The reagents used to prepare the synthetic CS were Fisher ACS grade MgCl_2_·6H_2_O, KH_2_PO_4_ and (NH_4_)_2_SO_4_, and distilled water. In addition, the same chemicals were used as the source of Mg and P for the RS and FS experiments to achieve the desired Mg:P:N molar ratio. Solutions of 1 N and 5 N NaOH and 1 N HCl were used to adjust the pH.

Physicochemical characteristics: The American Public Health Association (APHA, 1992) standard methods [33] were followed to measure the total solids (TS), volatile solids (VS), chemical oxygen demand (COD), and soluble COD. The COD was determined by the closed reflux colorimetric method. The orthophosphate concentration in the samples and in the supernatant was measured following the ascorbic acid method using a UV spectrophotometer. The pH value was measured using a pH meter (model, TIM840, Lognes, France). The total Kjeldahl nitrogen (TKN) and total ammonia nitrogen (TAN) were analysed using a Kjeltec auto-analyser (TECATOR 1030, Tecator AB, Hoganas, Sweden) following the macro-Kjeldahl method (APHA, 1992). All the heavy metals listed in Table 2, including Zinc (Zn), Copper (Cu), Lead (Pb), Nickel (Ni), Cadmium (Cd), Chromium (Cr), and Iron (Fe), were determined using the EPA method 3050 [34].

Precipitate characterization: The morphology of the precipitates was examined using scanning electron microscopy (SEM, FEI Helios Nanolab 660 DualBeam, Thermo Fisher Scientific, Waltham, MA, USA). To determine the crystalline structure of the precipitated powder, an X-ray diffractometer (XRD, Bruker Co., Yokohama, Japan, D8 Discovery X-ray Diffractometer, VANTEC Detector Cu-Source) was used.

Microbial community analysis: For the microbiological part, the liquid digestates from the three different 20-L digesters used (controls), the mix of digestates before struvite precipitation (named liquid + solid), and the processed samples (both the solid and liquid parts after solid–liquid separation) after the precipitation experiments were compared. DNA was extracted from the samples using a Qiagen All Prep DNA96 kit (Qiagen, Montreal, QC, Canada) with minor modifications. First, the samples (0.5 g) were mixed with 0.1 mm zirconia–silica beads (0.5 g; Fisher Scientific Ltd., St-Laurent, QC, Canada) and 0.5 mL of a tempered phenol/chloroform/isoamyl mixture (ratio 25:24:1 pH 8), and 0.5 mL of RLT+ (provided in the kit) were added per tube. The samples were lysed twice for 2 min using a Mini Beadbeater-8™ (BioSpec Products, Bartlesville, OK, USA) with 5 min cooling on ice in between. The aqueous phase containing nucleic acids was separated by centrifugation (16,000× *g*) for 15 min at 4 °C. A second extraction using phenol/chloroform/isoamyl (25:24:1 pH 8) was performed. The aqueous phases were combined with two volumes of the RLT+ lysis solution and the manufacturer’s instructions were followed to extract the DNA.

The samples were sequenced using the Genome Quebec (Montreal, QC, Canada) sequencing services with the Illumina Miseq PE250 technology, using the modified 515F and 806R [35] primers that target the 16S rRNA gene (V4–V5 regions). The sequences were analysed following the protocol discussed in [36] using the QIIME 2 bioinformatics platform. We used Cutadapt 2.10 to remove the primer and residual adaptor sequences. After the primer removal, the removal of the residual adaptor sequences was performed using the same parameters, but without the --p-discard-untrimmed option. The DADA2 program was used as a denoising method, and the reads were truncated at lengths of 227 and 222 bp for the forward and reverse reads, respectively. The Greengenes database version 2019 was used for the taxonomy analysis. The clustering was done using the Ward method in R. The RS before precipitation, the solid part and the liquid part samples, and the digestate had 94,438, 93,990, 99,018, and 62,289 sequences, respectively.

## 3. Results and Discussion

The study focused on struvite precipitation experiments using anaerobically digested chicken manure (CM) digestate samples, specifically a raw sample (RS) and a filtered sample (FS). Initially, the FS was employed to evaluate the potential impact of solids on the struvite precipitation process, as highlighted in the previous research [37]. This was then compared with a synthetically prepared controlled sample (CS) with known concentrations of nutrients.

The influence of suspended solids on the precipitation process is a key consideration in the integration of struvite precipitation with farm-scale anaerobic digestion. The cost of large-scale solids separation underlines the need to identify optimal conditions for struvite precipitation from the anaerobically digested CM digestate without resorting to large-scale solids separation methods. The following series of batch experiments were carried out using undiluted solutions (RS) under different operating conditions, as detailed in Table 1. This investigation aimed to assess the feasibility of incorporating struvite precipitation into the anaerobic digestion process without the necessity for substantial solid separation—a crucial aspect for practical applications at the farm scale.

### 3.1. Comparative Study: Filtered vs. Control Samples

To investigate the feasibility of struvite precipitation from the sample and to reduce the possible impact of the suspended solids in the precipitation process, a series of experiments were conducted using a filtered solution in various concentrations of digestates (FS1 to FS9) in comparison to the similar concentration of the control solution samples (CS1 to CS9). The rational for conducting the experiment using different concentrations of the digestate was to investigate whether a high concentration of nitrogen (N) in the digestate could hinder the precipitation or could lead to side product precipitation. For this comparison, the TAN removal efficiency was chosen as a reference parameter since most of the organically bounded N-content of the anaerobically digested CM digestate samples were mainly composed of TAN. Since our key objective was to investigate the feasibility of recovering N from the anaerobically digested CM digestate, the effects of the pH, dilution factor, and Mg:N:P molar ratio on the TAN recovery efficiency was investigated, as detailed in the subsequent sections. In this regard, the amount of TAN before and after each precipitation experiment was measured and compared, as outlined in Equation (1).

(1) $$\begin{array}{l} TAN\;Removal\;Efficiency\;(\%)\\ =\frac{{Initial\;TAN\;in\;Solution\left(mg/L\right)}_{\left(before\;precipitation\right)}-{Residual\;TAN\;in\;Supernatant\left(mg/L\right)}_{\left(after\;precipitation\right)}\;}{{Initial\;TAN\;in\;Solution\left(mg/L\right)}_{\left(before\;precipitation\right)}}\times 100 \end{array}$$

**Figure 1:**
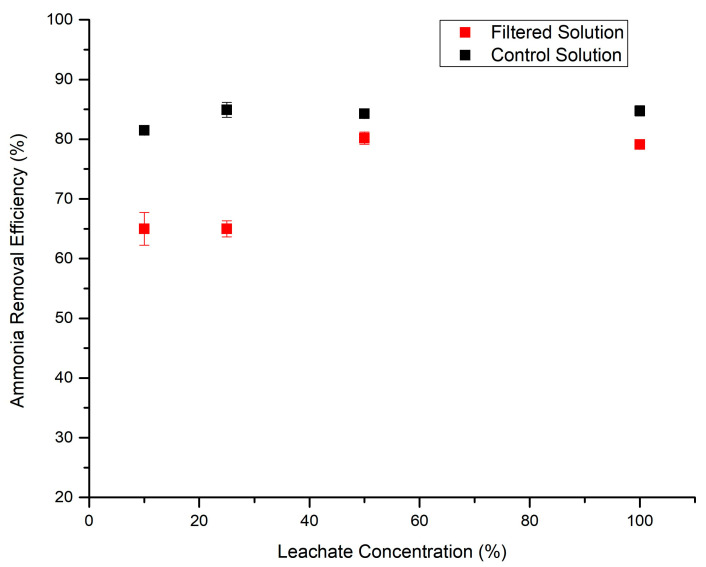
Impact of the digestate ammonia concentration on the ammonia removal efficiency through struvite precipitation.

Figure 1 shows the TAN removal efficiency of the various FSs and CSs with various digestate concentrations. As one can see, not only was struvite precipitation feasible with the CM digestate, the digestate samples with higher concentrations of ammonia (50% diluted and undiluted digestate) showed a higher ammonia removal efficiency and a very comparable trend with the control solution.

#### 3.1.1. Effect of pH on the TAN Removal Efficiency by Struvite

**Figure 2:**
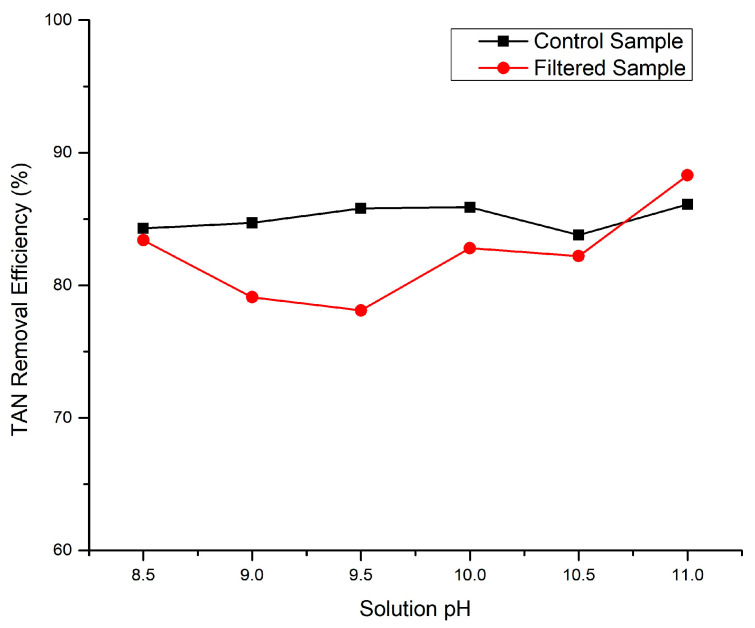
Total ammonia nitrogen removal efficiency in different solution pHs for the control samples (CS4 to CS9) and filtered samples (FS4 to FS9).

Figure 2 illustrates the effect of the pH on the efficiency of TAN removal through struvite precipitation. The pH range examined was from 8.5 to 11, which was reported as the most probable range for struvite precipitation [38]. Although the pH did not exert a dominant influence on struvite precipitation in this instance, a pH of 11 exhibited the most notable performance in TAN removal, achieving 86.1% and 88.3% in the control and filtered samples, respectively. It is crucial to acknowledge that the optimal pH for struvite precipitation can vary based on wastewater composition and the characteristics of the solid waste under treatment. In this particular scenario, a pH of 11 demonstrated the highest efficiency in total ammonia nitrogen removal. This outcome was likely attributable to the favorable conditions for struvite precipitation at this pH level. The heightened concentration of hydroxide ions at higher pHs facilitates the binding of magnesium (Mg^2+^), ammonium (NH_4_^+^), and phosphate (PO_4_^3−^) ions, leading to the formation of struvite crystals and effectively eliminating ammonia from the solution.

#### 3.1.2. Effect of Digestate Dilution on the Precipitation Yield

**Figure 3:**
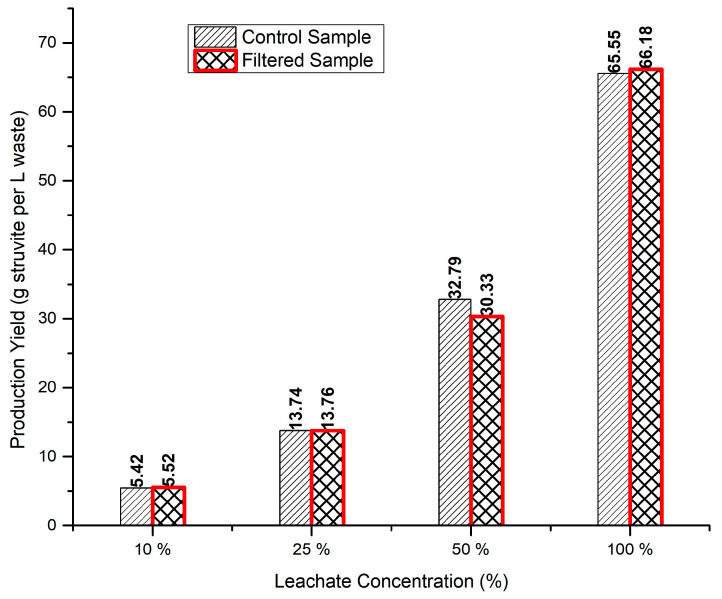
Struvite production yields of the filtered sample and control sample with different digestate concentrations.

Another important comparative analysis in this work was to study the impact of the initial ammonia concentrations on TAN removal and consequently on the precipitation yield (grams of struvite produced). This could be a crucial factor for scaling up the process if the undiluted digestate shows a limited precipitation yield. In this case, to achieve a successful struvite precipitation process, the CM–AD would need to be diluted with additional water to reach the desired initial nitrogen concentration. This dilution will create new challenges, such as larger size digesters and higher water volumes. Eight separate samples of CS and FS solutions with different initial TAN concentrations (CS1 to CS4 and FS1 to FS4) with digestate concentrations of 10, 25, 50, and 100%, respectively, were prepared to conduct the precipitation experiment. The precipitates were then filtered, dried, and accurately weighed. As shown in Figure 3, there was no major discrepancy in the precipitation yields between the control and filtered samples for each pair of samples with similar initial TAN concentrations. This may have demonstrated the limited effect of foreign ions in the filtered sample on the precipitation yield compared to the control sample, in which there were no foreign ions.

On the other hand, increasing the initial concentration of TAN from 10% to 100% not only did not limit the precipitation yield, but also increased the efficiency of TAN removal substantially, from 65% in the FS1 experiment to 80% in the FS4 solution. This could have been the result of multiple factors. The two most important factors for this significant increase in the struvite formation are as follows. (i) A higher initial leachate concentration will simply increase the main driving force for precipitation. In a solution with a higher initial ammonia concentration, the driving force for struvite crystal formation is greater. Struvite precipitation occurs when the concentrations of magnesium (Mg^2+^), ammonium (NH_4_^+^), and phosphate (PO_4_^3−^) exceed their solubility limits. A higher initial concentration of TAN provides more reagents for struvite crystal formation. (ii) Under chemical conditions similar to this experiment, there may be competing reactions or ions that interfere with struvite precipitation. A higher initial concentration of TAN in the leachate can overcome competing reactions, leading to more selective struvite formation.

### 3.2. Struvite Precipitation from the Raw Sample

After the initial comparative study involving the filtered and control samples, the subsequent experiments were carried out using the undiluted raw solution under various experimental conditions, as detailed in Table 1 (RS 1 to RS 9). The utilization of lower concentrations of the RS solutions (i.e., 10%, 25%, 50%) was deemed unnecessary, as the undiluted raw solution (100% raw solution) exhibited the anticipated struvite precipitation rate and an ammonia removal efficiency comparable to the CS and FS solutions.

#### 3.2.1. Effect of pH on TAN Removal Efficiency

**Figure 4:**
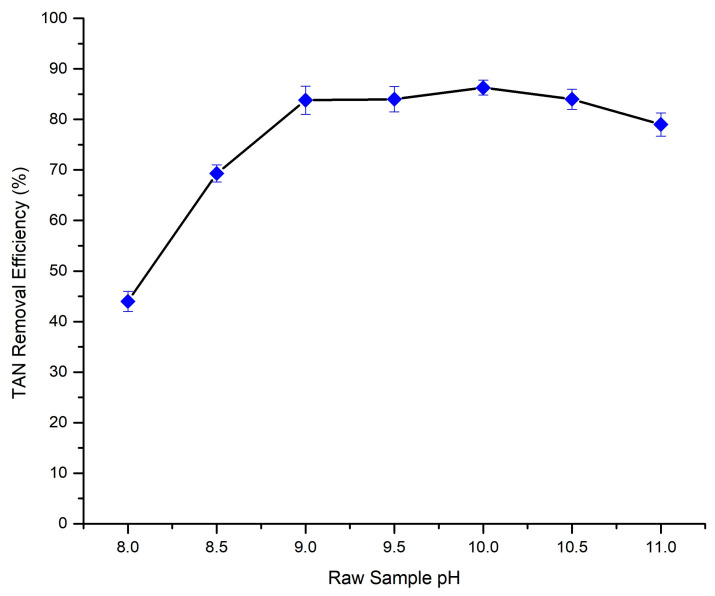
Total ammonia nitrogen removal efficiency of the raw sample at different pHs (undiluted digestate and Mg:P:N of 1:1:1).

Figure 4 illustrates the impact of the pH on the total ammonia removal efficiency through struvite precipitation of the raw samples within the pH range of 8 to 11. At a Mg:P:N molar ratio of 1:1:1, it was observed that the total ammonia removal was relatively low at pH 8 (44%) and increased significantly with a rise in the pH. This increase demonstrated a remarkable progression, escalating from 44% at pH 8 to 69.3% at pH 8.5, and further to 83.3% at pH 9. Beyond pH 9, the change in the TAN removal efficiency became less pronounced, decreasing to 79% at pH 11. Notably, pH 10 exhibited the highest removal efficiency at 86.3%, establishing it as the optimal pH for the subsequent parametric analysis of the RS samples.

#### 3.2.2. Effect of the Mg:P:N Molar Ratio on the TAN Removal Efficiency

**Figure 5:**
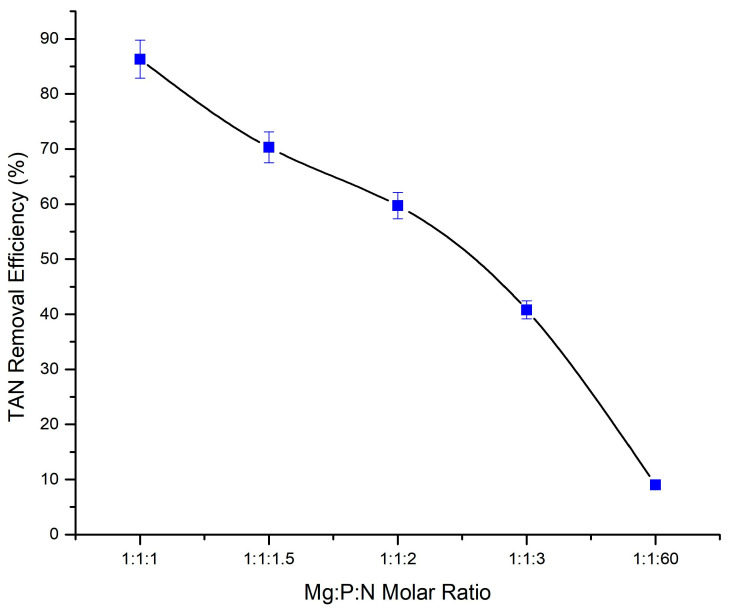
Total ammonia nitrogen removal in the undiluted raw sample at different Mg:P:N molar ratios (at pH 10).

The formation of struvite requires equimolar amounts of phosphate and ammonium. However, the initial Mg:P:N molar ratio in the CM–AD raw digestate was notably imbalanced at approximately 1:1:60. Consequently, the total ammonia nitrogen removed by struvite precipitation in the experiments without additional phosphate and magnesium was limited. To enhance TAN removal, the initial molar ratio was adjusted by introducing phosphate and magnesium sources before each experiment (FS5, FS8, FS9, and FS10). This adjustment aimed to achieve molar ratios of 1:1:1, 1:1:1.5, 1:1:2, and 1:1:3, respectively. The data shown in Figure 5, depicting the impact of the Mg:P:N molar ratios on the TAN removal efficiency at pH 10, revealed a significant increase from around 9% to 86.3% as the initial ratio improved from 1:1:60 to the optimal 1:1:1. However, a decline in efficiency was observed with lower ratios (1:1:1.5, 1:1:2, and 1:1:3). This emphasized the significance of maintaining a 1:1:1 molar ratio for optimal TAN recovery through the struvite precipitation process, aligning with the previous studies [29,39]. The findings highlighted the importance of carefully adjusting the Mg:P:N ratios to improve the efficiency of TAN removal through struvite precipitation, a critical consideration for the efficient recovery of nitrogen from the anaerobically digested chicken manure digestate.

### 3.3. Particle Characterization

In the quest to validate the presence of struvite, the particles derived from the anaerobically digested CM digestate underwent meticulous characterization using two distinct methods: XRD diffraction and SEM imaging. This comprehensive analysis aimed to confirm the struvite identity and shed light on the crystalline and morphological attributes of the precipitates. The precipitate sample chosen for this investigation was obtained from the raw sample at pH 10 (experiment RS5), which demonstrated the highest nitrogen removal efficiency, and thus was deemed the recommended operational condition for potential full-scale applications.

**Figure 6:**
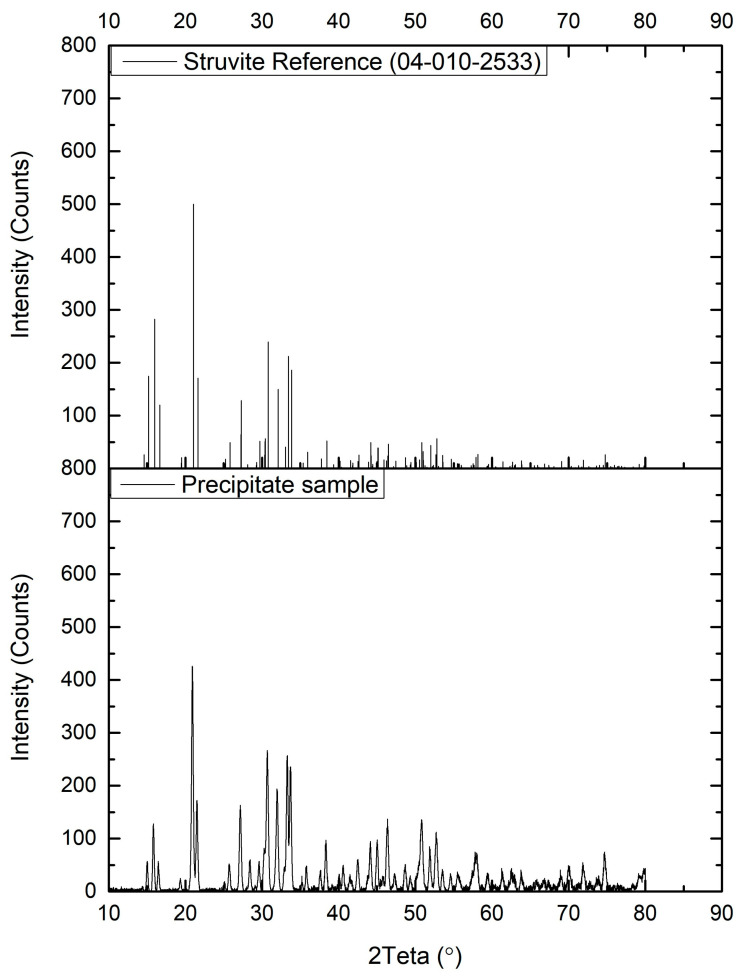
XRD pattern of struvite obtained from the raw solution at pH 10 and a 1:1:1 molar ratio of Mg, P and N.

Figure 6 illustrates the XRD diffraction results from the RS5 experiment, showcasing a striking resemblance to the standard struvite XRD peaks derived from the International Center for Diffraction Data. Despite subtle fluctuations in the peaks, suggesting the presence of co-precipitates, the predominant diffraction pattern unequivocally pointed towards the crystalline nature of struvite. The consistent alignment with struvite peaks affirmed the successful formation of struvite crystals in the anaerobically digested CM digestate under the specified experimental conditions.

**Figure 7:**
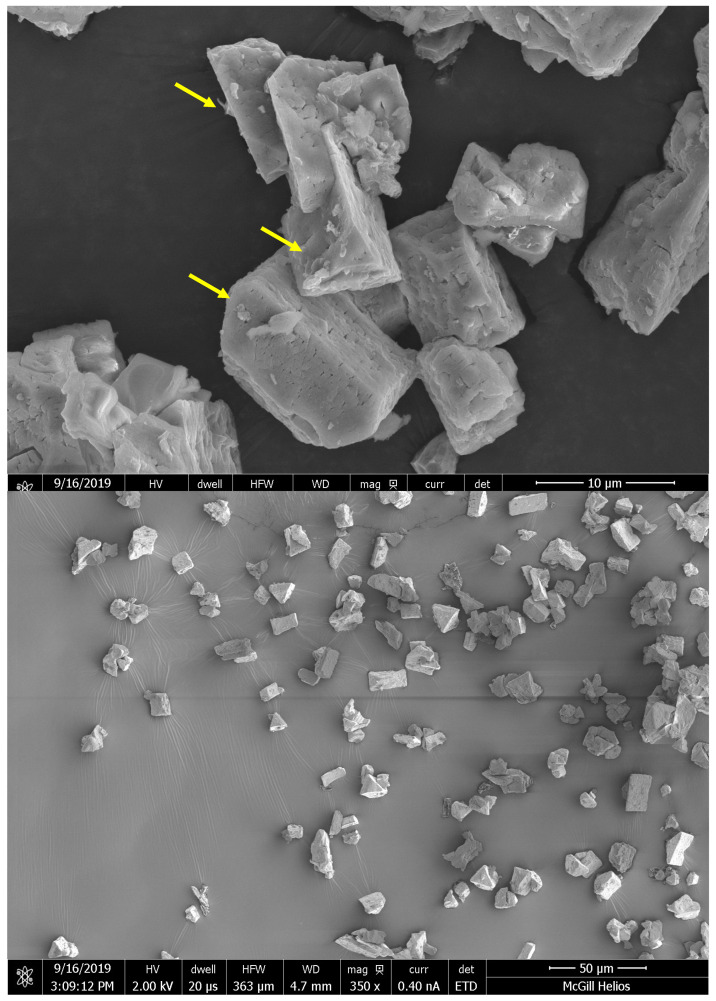
SEM micrograph of struvite obtained from the raw solution at pH 10 and a 1:1:1 molar ratio at different magnifications.

Similarly, the SEM analysis of the RS5 precipitate sample further corroborated the predominance of struvite crystals. Struvite is known for its stable white crystals with an orthorhombic pyramidal crystal lattice [15,24]. Figure 7 vividly portrays the crystalline structure of the precipitated particles, predominantly exhibiting an orthorhombic arrangement and further affirming their struvite composition (highlighted by yellow arrows in Figure 7). While the SEM images may not precisely convey the particle size distribution due to the collection and drying methods employed during quantification, they serve as crucial visual evidence, confirming the distinctive structure of the struvite crystals.

This detailed particle characterization not only provided evidence of successful struvite formation in the anaerobically digested CM digestate but also contributed valuable insights into the potential scalability of the struvite precipitation process. The selection of the recommended operational condition, based on the RS5 experiment, considered both the nitrogen removal efficiency and the crystalline characteristics of struvite. These findings are pivotal for advancing the understanding of struvite precipitation dynamics in complex anaerobic digestion matrices, guiding future endeavors toward sustainable nitrogen recovery practices.

### 3.4. Effect of Solids on the Struvite Precipitation Yield

**Figure 8:**
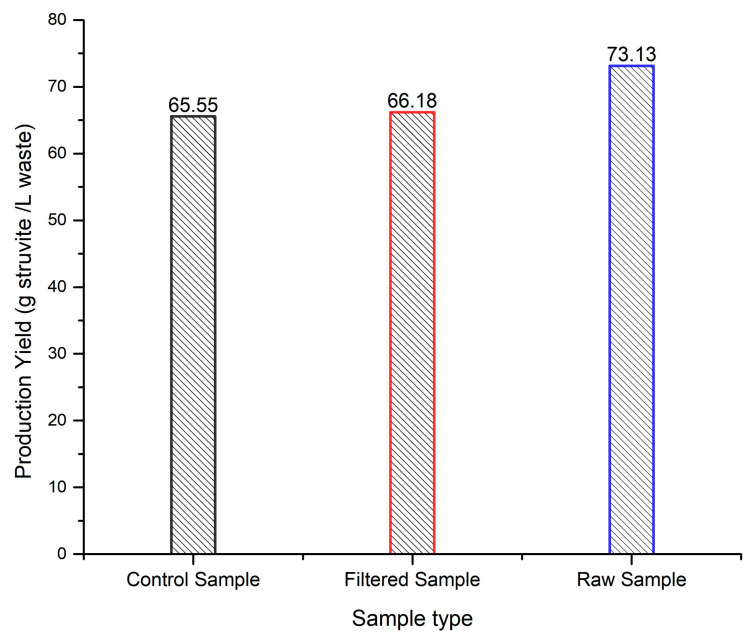
Struvite precipitation yield of the different undiluted samples, CS, FS, and RS, under similar experiment conditions (pH 10 and a 1:1:1 molar ratio).

Struvite precipitation from the CM–AD, characterized by a complex chemical matrix, poses potential challenges, notably with the substantial presence of solids in the raw sample. This study systematically assessed the influence of solids on the struvite precipitation yield. Surprisingly, the data shown in Figure 8 revealed that the presence of solids in the raw sample not only did not prevent struvite formation but, counterintuitively, enhanced the overall production yield of the process. The increase in the precipitate amount in the raw sample compared to the filtered sample (73.13 g/L of waste vs. 66.18 g/L of waste) suggested a potential mechanism wherein suspended solids become entrapped in the crystal matrix, simultaneously leading to the partial removal of suspended solids. This unanticipated finding emphasized the resilience of the struvite precipitation process in the presence of a complex chemical matrix and underscored the potential for an increased yield with the inclusion of solids, highlighting an intriguing aspect for further exploration in optimizing struvite recovery from the anaerobically digested chicken manure digestate.

### 3.5. Impact on Heavy Metal Removal

Table 3 presents the initial amount of the selected metal elements present in the anaerobically digested CM digestate samples (sample RS5) and their corresponding concentrations after being subjected to struvite precipitation. Specifically, the concentrations of heavy metals, including Zn, Cu, Pb, Ni, Cd, Cr, and Fe in the CM–AD, were 5.56, 6.63, 0.01, 0.48, 0.02, 0.10, and 10.70 mg/L, respectively, prior to struvite precipitation. These concentrations were significantly reduced to 0.54, 0.45, 0.007, 0.31, 0.007, 0.007, and 2.92 mg/L after treatment using the struvite precipitation technique, resulting in removal efficiencies of 90.2%, 93.2%, 36.4%, 35.7%, 56.2%, 93%, and 72.7%, respectively. Two different scenarios may have accounted for this significant reduction. These metal elements were adsorbed onto the surface of struvite crystals or co-precipitated with struvite crystals [31]. In both cases, the reduction in heavy metals in the digestate (supernatant) rendered it harmless for discharge into the environment.

It’s worth mentioning that heavy metals can be incorporated into struvite crystals or precipitates through co-precipitation, where certain heavy metal ions are incorporated into the crystal lattice of the precipitating struvite. These encapsulated heavy metals are effectively immobilized in the struvite solid structure. Although struvite is generally stable, the conditions or changes in the environment can lead to the release of trapped heavy metals. For example, changes in the pH, redox potential, or the presence of certain chemical agents could potentially affect the stability of struvite crystals and lead to the release of heavy metals. It is important to properly dispose of or reuse precipitated struvite as fertilizer to avoid any potential leaching or the release of heavy metals in the future. To understand the fate of heavy metals, thorough monitoring and analysis is essential. This involves analysing the composition and stability of the struvite crystals, as well as monitoring the leachate for any potential release of heavy metals over time. Further studies are warranted to investigate the fate of these heavy metals.

### 3.6. Microbial Community Behaviour

**Figure 9:**
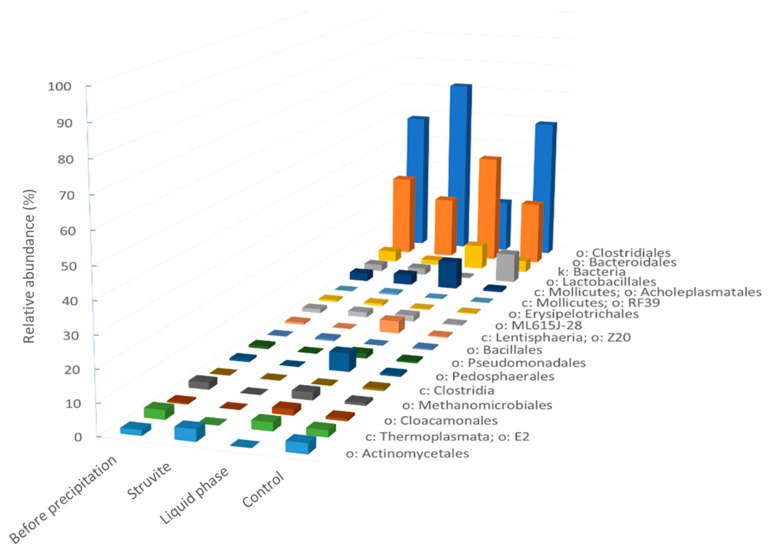
Relative abundances of the most abundant bacterial groups in each sample type. The control samples were digestates from the three digesters. The letters denote the following: k: kingdom; o: order; and c: class.

As shown in Figure 9, the predominant microbial orders were *Clostridiales* and *Bacteroidales* in all the samples. Their combined relative abundance represented more than 50% in all the samples. The liquid sample was mostly dominated by *Bacteroidales* whereas the whole struvite sample, the solid part (struvite), and the control samples contained more *Clostridiales* than *Bacteroidales.*

**Figure 10:**
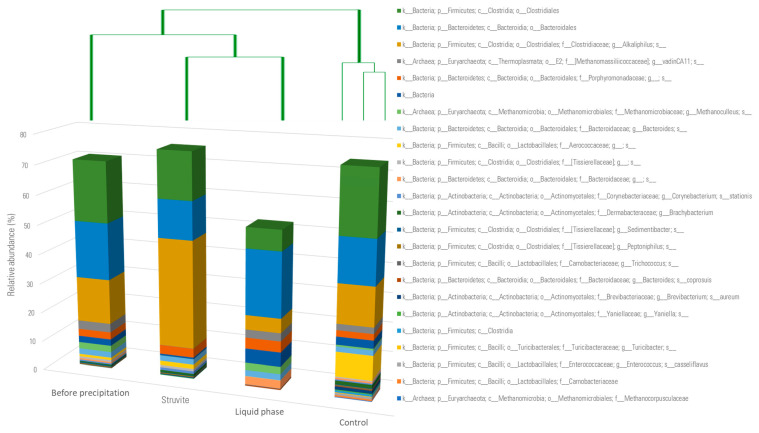
Relatedness of the microbial communities using the clustering Ward method. The relative abundance of the major amplicon sequence variants (ASVs) are shown above. The control samples were digestates from each of the three digesters. The letters denote the following: k: kingdom; c: class; o: order; f: family; g: genus; and s: species.

Figure 9 shows the relative abundance of the major taxa while Figure 10 considers all the amplicon sequence variants (ASVs). The most abundant *Clostridiales* in each sample was the *Alkaliphilus* genus (Figure 10), which had a relative abundance of 15% in the whole struvite sample before precipitation, 37% in the struvite sample, 5% in the liquid phase, and 13% in the control (digestates). This genus was identified in a methanogenic consortia from anaerobic digesters under high ammonia stress [40]. The *Thermoplasmata* were mainly composed of the *Methanomassiliicoccaceae* genus vadinCA11, which is an ammonia-tolerant methanogen. The *Methanomicrobiales* were less abundant in the struvite sample (0.06%) and in the control (0.8%) when compared to the sample before precipitation (2.30%) and the liquid phase after precipitation (2.5%). Thus, struvite precipitation would not lead to the removal of these methanogens in the liquid phase. *Methanoculleus* was the most abundant member of *Methanomicrobiales* (Figure 10) and is typically found in digesters fed with manure [41,42]. The liquid phase had the highest relative abundance of *Acholeplasmatales*, *Cloacamonales*, *Pedosphaerales*, *Pseudomonadales*, and *Lentisphaeria* compared to the whole struvite sample before precipitation, the struvite, and the control samples.

The clustering analysis revealed that the control samples, which were digestates from the three digesters, had a different microbial community when compared to the other samples related to struvite precipitation (Figure 10). The microbial community of the solid part was more similar to that of the sample before precipitation than to the microbial community of the liquid part. These findings confirmed that the struvite precipitation process affected the microbiological community.

### 3.7. Process Optimization and Operational Consideration

This study assessed the viability of using struvite precipitation as a post-treatment method for nutrient recovery after anaerobic digestion of chicken manure. Various parameters, including the pH, initial ammonia concentration, molar ratios of constituent ions, and the potential impact of solids on precipitation, were investigated. The total ammonia nitrogen (TAN) removal efficiency served as a key performance indicator in all the experiments. Precipitation experiments conducted on the filtered samples demonstrated the effectiveness of this technique in recovering nutrients, particularly nitrogen, from the anaerobically digested chicken manure digestate (CM–AD). Despite the complex chemical composition of the chicken manure digestate, the experiments conducted at a pH of 10 showed the highest TAN recovery efficiency, at 86.3% for the raw sample. Notably, the precipitation experiments with the filtered samples mirrored the trends observed in the experiments with the control samples, underlining the consistency of the TAN removal efficiency and struvite production yield. This underscores the capability of the struvite precipitation technique for effectively recovering nutrients from anaerobically digested chicken manure, emphasizing its potential as a valuable nutrient recovery strategy in waste management.

As this study aimed to provide solutions for the integration of struvite precipitation with anaerobic digestion, particularly suitable for farm-scale operations, we investigated this process using raw samples to eliminate the costs and resources required for the liquid–solid separation step. Interestingly, the results of the raw sample experiment were very promising in terms of the process feasibility and performance. The parametric studies determined that the pH range of 9 to 10 was the most suitable for struvite precipitation in the raw sample of the anaerobically digested CM digestate, as previous studies have indicated as the optimum pH for struvite recovery from different types of animal manure [24,28,31]. Furthermore, the effect of the Mg:P:N molar ratio on the TAN removal efficiency was studied, and an almost inverse linear correlation was observed between these two elements. As shown in Figure 4, by increasing the molar ratio of N to P and Mg, the TAN removal efficiency decreased almost proportionally. For the Mg:P:N molar ratios of 1:1:2 and 1:1:3, the TAN removal efficiency decreased from 86.3% to 59.7% and 40.8%, respectively. This decrease was expected to be greater. According to stochiometric calculations, the elimination efficiency associated with the 1:1:3 ratio, for example, should have been around 30%. However, a greater ammonia removal was observed. This could have been due to the availability of free ammonia in the solution for stripping in the air, compared to the 1:1:1 molar ratio which had a significantly lower amount of free ammonia in the solution for stripping in the air. Determining this correlation between the Mg:P:N molar ratio and TAN removal efficiency will provide a process optimization parameter for large-scale operations that can be used to remove only the desired amount of TAN from the digestate while reducing the cost of adding additional Mg and P sources.

One potential impact of integrating the struvite precipitation technique with anaerobic digestion could be the removal of heavy metals from the AD digestate, which has been reported before [43,44]. These metallic elements, originating from chicken feed or feed additives, could lead to soil contamination if the digestate from chicken manure is directly applied to agricultural lands [11]. Considering the fact that the heavy metals total contents are not altered during anaerobic digestion, this integration offers dual benefits in terms of heavy metal removal. Consequently, the final effluent becomes more eco-friendly and can be discharged into the environment or reused in non-potable applications, including agricultural activities. While this study demonstrated some removal of heavy metals, ongoing research is being conducted to ascertain whether these metals have been trapped in the struvite crystals or eliminated through the precipitation process. Further investigations will provide a comprehensive understanding of the mechanisms involved in heavy metal removal and contribute to refining the integration of struvite precipitation with anaerobic digestion for enhanced environmental sustainability.

## 4. Conclusions

This study offers valuable insights into the practical application of the struvite precipitation process to enhance nutrient recovery from the digestate following anaerobic digestion. Notably, the study revealed that the initial ammonia concentration in the chicken manure digestate had a minimal impact on the overall efficiency of the precipitation process. The comparable results between the filtered and control samples suggested that digestate dilution may not be necessary in large-scale operations, emphasizing a cost-effective approach. Optimal pH conditions for nitrogen recovery were identified at pH 11, demonstrating substantial ammonia removal efficiencies of 86% and 88% for the control and filtered samples, respectively. However, it should be noted that pH 10 proved effective for TAN removal in the raw samples, introducing a pH-specific consideration in the practical implementation of struvite precipitation.

Furthermore, the study highlighted significant heavy metal reductions up to 93% in leachate due to struvite precipitation and an improved microbial community composition in the digestate. While promising, it is essential to acknowledge the limitations of this study. The specific mechanisms behind heavy metal removal and whether these metals are trapped in the struvite crystals or eliminated entirely require further investigation. Despite these limitations, the struvite precipitation technique improved nutrient recovery and is a promising method for treating chicken manure, aligning with environmentally sustainable practices and resource recovery goals.

## Figures and Tables

**Table 1:** List of the respective experimental conditions.

Sample	Test	Digested CM Concentration	pH	Mg:P:N Molar Ratio	Initial Total Ammonical Nitrogen (TAN) (mg/L)
Control Sample	CS1	10%	9	1:1:1	324
	CS2	25%	9	1:1:1	810
	CS3	50%	9	1:1:1	1620.5
	CS4	100%	8.5	1:1:1	3241
	CS5	100%	9	1:1:1	3241
	CS6	100%	9.5	1:1:1	3241
	CS7	100%	10	1:1:1	3241
	CS8	100%	10.5	1:1:1	3241
	CS9	100%	11	1:1:1	3241
Filtered Sample	FS1	10%	9	1:1:1	393.9
	FS2	25%	9	1:1:1	984.7
	FS3	50%	9	1:1:1	1969.5
	FS4	100%	8.5	1:1:1	3939
	FS5	100%	9	1:1:1	3939
	FS6	100%	9.5	1:1:1	3939
	FS7	100%	10	1:1:1	3939
	FS8	100%	10.5	1:1:1	3939
	FS9	100%	11	1:1:1	3939
Raw Sample	RS1	100%	8	1:1:1	3979
	RS2	100%	8.5	1:1:1	3979
	RS3	100%	9	1:1:1	3979
	RS4	100%	9.5	1:1:1	3979
	RS5	100%	10	1:1:1	3979
	RS6	100%	10.5	1:1:1	3979
	RS7	100%	11	1:1:1	3979
	RS8	100%	10	1:1:1.5	3979
	RS9	100%	10	1:1:2	3979
	RS10	100%	10	1:1:3	3979

**Table 2:** Physicochemical characteristics of the raw and filtered samples of anaerobically digested chicken manure (digestate).

Parameters	Symbol	Unit	Raw Sample	Filtered Sample
Total Kjeldahl nitrogen	TKN	mg/L	4863 ± 71	4435 ± 49
Total ammonical nitrogen	TAN	mg/L	4036 ± 34	3939 ± 37
Total solid	TS	weight %	2.16 ± 0.06	1.63 ± 0.04
Volatile solid	VS	weight %	1.01 ± 0.02	0.64 ± 0.01
Total COD	CODt	mg/L	18,262 ± 109	11,394 ± 89
Soluble COD	CODs	mg/L	11,541 ± 76	11,282 ± 55
Total phosphorus	TP	mg/L	195 ± 19	58.70 ± 11
Orthophosphate	PO4-P	mg/L	71.10 ± 6.10	43.70 ± 4.91
Potassium	K	mg/L	4160 ± 39	4140 ± 33
Calcium	Ca	mg/L	322 ± 27	13.70 ± 2.5
Magnesium	Mg	mg/L	58.70± 4.32	5.11 ± 0.91
Sodium	Na	mg/L	765 ± 61	761 ± 57
Zinc	Zn	mg/L	5.20 ± 1.08	0.47 ± 0.02
Copper	Cu	mg/L	6.36 ± 1.19	0.48 ± 0.01
Lead	Pb	mg/L	0.01	<0.01
Nickel	Ni	mg/L	0.42	0.35
Silver	Ag	mg/L	<0.0050	<0.0050
Aluminum	Al	mg/L	4.36 ± 0.23	3.05 ± 0.22
Arsenic	As	mg/L	0.02	0.020
Iron	Fe	mg/L	10.70 ± 1.99	9.34 ± 1.41
Silicon	Si	mg/L	43.50 ± 3.5	42.10 ± 3.3
Boron	B	mg/L	3.60 ± 0.42	3.56 ± 0.33
Barium	Ba	mg/L	0.30	0.06
Chromium	Cr	mg/L	0.10	0.19
Mercury	Hg	mg/L	0.001	0.0005
Lithium	Li	mg/L	0.24	0.24
Manganese	Mn	mg/L	3.62 ± 0.67	0.14 ± 0.02
Tin	Sn	mg/L	<0.050	<0.050
Titanium	Ti	mg/L	0.10	0.10
Vanadium	VS	mg/L	0.03	0.03

**Table 3:** Concentration of the various heavy metal elements before and after struvite precipitation.

Element	Unit	CM–AD Digestate (Prior to Struvite Precipitation Experiment)	Supernatant (After Struvite Precipitation Experiment)	Removal Efficiency (%)
Zinc (Zn)	mg/L	5.56	0.54	90.20
Copper (Cu)	mg/L	6.63	0.45	93.20
Lead (Pb)	mg/L	0.01	0.007 *	36.40
Nickel (Ni)	mg/L	0.48	0.31	35.70
Cadmium (Cd)	mg/L	0.02	0.007 *	56.20
Chromium (Cr)	mg/L	0.10	0.007 *	93.00
Iron (Fe)	mg/L	10.70	2.92	72.70

* Detectable limit.

## Data Availability

The data may be available from authors upon reasonable request.

## References

[1] Faostat F. (2016). FAOSTAT Statistical Database.

[2] Alexandratos N., Bruinsma J. (2012). World Agriculture towards 2030/2050: The 2012 Revision.

[3] Sims J.T. (1997). Agricultural and Environmental Issues in the Management of Poultry Wastes: Recent Innovations and Long-Term Challenges.

[4] Rajagopal R., Massé D.I. (2016). Start-up of dry anaerobic digestion system for processing solid poultry litter using adapted liquid inoculum. Process. Saf. Environ. Prot..

[5] Miller B. (1984). Extruding Hatchery Waste. Poult. Sci..

[6] Bernal M., Alburquerque J., Moral R. (2009). Composting of animal manures and chemical criteria for compost maturity assessment. A review. Bioresour. Technol..

[7] Sobel A.T., Ludington D.C. (1966). Destruction of chicken manure by incineration. Management of Farm Animal Wastes. Proc. Nat. Symp. on Animal Waste Management.

[8] Rajagopal R., Massé D.I., Singh G. (2013). A critical review on inhibition of anaerobic digestion process by excess ammonia. Bioresour. Technol..

[9] Tao W., Ukwuani A.T. (2015). Coupling thermal stripping and acid absorption for ammonia recovery from dairy manure: Ammonia volatilization kinetics and effects of temperature, pH and dissolved solids content. Chem. Eng. J..

[10] Limoli A., Langone M., Andreottola G. (2016). Ammonia removal from raw manure digestate by means of a turbulent mixing stripping process. J. Environ. Manag..

[11] Nkoa R. (2013). Agricultural benefits and environmental risks of soil fertilization with anaerobic digestates: A review. Agron. Sustain. Dev..

[12] Nagarajan A., Goyette B., Raghavan V., Bhaskar A., Rajagopal R. (2023). Nutrient recovery via struvite production from livestock ma-nure-digestate streams: Towards closed loop bio-economy. Process. Saf. Environ. Prot..

[13] Seruga P., Krzywonos M., Pyżanowska J., Urbanowska A., Pawlak-Kruczek H., Niedźwiecki Ł. (2019). Removal of Ammonia from the Municipal Waste Treatment Effluents using Natural Minerals. Molecules.

[14] Lunt O.R., Kofranek A.M., Clark S.B. (1964). Nutrient Availability in Soil, Availability of Minerals from Magnesium Ammonium Phosphates. J. Agric. Food Chem..

[15] Lee J., Rahman M., Ra C. (2009). Dose effects of Mg and PO4 sources on the composting of swine manure. J. Hazard. Mater..

[16] Barker J.C., Hodges S.C., Walls F.R. (2002). “Livestock Manure Production Rates and Nutrient Content. Chapter 10-Fertilizer Use”, in 2002 North Carolina Agricultural Chemicals Manual. https://www.yumpu.com/en/document/read/35203230/livestock-manure-production-rates-and-nutrient-content-ontario-agri.

[17] Mousavi S.E. (2017). Modeling Struvite Precipitation in a Batch Reactor Using a Population Balance Model in a 3-D Computational Fluid Dynamic (CFD) Framework. Master’s Thesis.

[18] Thompson L.B. (2013). Field Evaluation of the Availability for Corn and Soybean of Phosphorus Recovered as Struvite from Corn Fiber Processing for Bioenergy. Master’s Thesis.

[19] Gell K., Ruijter F., Kuntke P., de Graaff M., Smit A. (2011). Safety and Effectiveness of Struvite from Black Water and Urine as a Phosphorus Fertilizer. J. Agric. Sci..

[20] Pérez R.C., Steingrobe B., Romer W., Claassen N. (2009). Plant Availability of Pfertilizers Recycled from Sewage Sludge and Meat-and-Bone Meal in Field and Pot Experiments.

[21] Mousavi S.E., Choudhury M.R., Rahaman M.S. (2019). 3-D CFD-PBM coupled modeling and experimental investigation of struvite precipi-tation in a batch stirred reactor. Chem. Eng. J..

[22] Tarragó E., Puig S., Ruscalleda M., Balaguer M.D., Colprim J. (2016). Controlling struvite particles’ size using the up-flow velocity. Chem. Eng. J..

[23] Li B., Boiarkina I., Yu W., Huang H.M., Munir T., Wang G.Q., Young B.R. (2018). Phosphorous recovery through struvite crystallization: Challenges for future design. Sci. Total Environ..

[24] Tao W., Fattah K.P., Huchzermeier M.P. (2016). Struvite recovery from anaerobically digested dairy manure: A review of application potential and hindrances. J. Environ. Manag..

[25] Zhang H., Lo V.K., Thompson J.R., Koch F.A., Liao P.H., Lobanov S., Mavinic D.S., Atwater J.W. (2014). Recovery of phosphorus from dairy manure: A pilot-scale study. Environ. Technol..

[26] Jordaan E.M., Ackerman J., Cicek N. (2010). Phosphorus removal from anaerobically digested swine wastewater through struvite precipitation. Water Sci. Technol..

[27] Song Y.-H., Qiu G.-L., Yuan P., Cui X.-Y., Peng J.-F., Zeng P., Duan L., Xiang L.-C., Qian F. (2011). Nutrients removal and recovery from anaerobically digested swine wastewater by struvite crystallization without chemical additions. J. Hazard. Mater..

[28] Cerrillo M., Palatsi J., Comas J., Vicens J., Bonmatí A. (2015). Struvite precipitation as a technology to be integrated in a manure anaerobic digestion treatment plant–removal efficiency, crystal characterization and agricultural assessment. J. Chem. Technol. Biotechnol..

[29] Yetilmezsoy K., Sapci-Zengin Z. (2009). Recovery of ammonium nitrogen from the effluent of UASB treating poultry manure wastewater by MAP precipitation as a slow release fertilizer. J. Hazard. Mater..

[30] Yilmazel Y., Demirer G. (2011). Removal and recovery of nutrients as struvite from anaerobic digestion residues of poultry manure. Environ. Technol..

[31] Muhmood A., Wu S., Lu J., Ajmal Z., Luo H., Dong R. (2018). Nutrient recovery from anaerobically digested chicken slurry via struvite: Performance optimization and interactions with heavy metals and pathogens. Sci. Total Environ..

[32] Mahato P., Rajagopal R., Goyette B., Adhikary S. (2022). Low-temperature anaerobic digestion of chicken manure at high organic and nitrogen loads—Strategies for controlling short chain fatty acids. Bioresour. Technol..

[33] American Public Health Association (1926). Standard Methods for the Examination of Water and Wastewater.

[34] U.S. Environmental Protection Agency (1996). Method 3050B: Acid Digestion of Sediments, Sludges, and Soils.

[35] Walters W., Hyde E.R., Berg-Lyons D., Ackermann G., Humphrey G., Parada A., Gilbert J.A., Jansson J.K., Caporaso J.G., Fuhrman J.A. (2016). Improved Bacterial 16S rRNA Gene (V4 and V4-5) and Fungal Internal Transcribed Spacer Marker Gene Primers for Microbial Community Surveys. mSystems.

[36] Estaki M., Jiang L., Bokulich N.A., McDonald D., González A., Kosciolek T., Martino C., Zhu Q., Birmingham A., Vázquez-Baeza Y. (2020). QIIME 2 Enables Comprehensive End-to-End Analysis of Diverse Microbiome Data and Comparative Studies with Publicly Available Data. Curr. Protoc. Bioinform..

[37] Tarragó E., Sciarria T.P., Ruscalleda M., Colprim J., Balaguer M.D., Adani F., Puig S. (2018). Effect of suspended solids and its role on struvite formation from digested manure. J. Chem. Technol. Biotechnol..

[38] Le Corre K.S., Valsami-Jones E., Hobbs P., Parsons S.A. (2009). Phosphorus Recovery from Wastewater by Struvite Crystallization: A Review. Crit. Rev. Environ. Sci. Technol..

[39] Yang H.R., Zhang Y.L., Zhou U.F., Zhu H.G. (2012). Recovering Ammonium-Nitrogen and Phosphorus through Struvite from Anaerobic Digested Effluent of Poultry Wastewater: Effects of Reagent Ratio and pH. Adv. Mater. Res..

[40] Lee J., Han G., Shin S.G., Koo T., Cho K., Kim W., Hwang S. (2016). Seasonal monitoring of bacteria and archaea in a full-scale thermophilic anaerobic digester treating food waste-recycling wastewater: Correlations between microbial community characteristics and process variables. Chem. Eng. J..

[41] Liu L., Du Z., Li Y., Han R. (2023). Study on Anaerobic Digestion Characteristics of Hulless Barley Straw and Livestock Manure. Biotechnol. Bioprocess Eng..

[42] Krause L., Diaz N.N., Edwards R.A., Gartemann K.-H., Krömeke H., Neuweger H., Pühler A., Runte K.J., Schlüter A., Stoye J. (2008). Taxonomic composition and gene content of a methane-producing microbial community isolated from a biogas reactor. J. Biotechnol..

[43] Uysal A., Demir S., Sayilgan E., Eraslan F., Kucukyumuk Z. (2013). Optimization of struvite fertilizer formation from baker’s yeast wastewater: Growth and nutrition of maize and tomato plants. Environ. Sci. Pollut. Res..

[44] Liu Y., Kwag J.-H., Kim J.-H., Ra C. (2011). Recovery of nitrogen and phosphorus by struvite crystallization from swine wastewater. Desalination.

